# Characterization and bioactivities of glutaminase-free L-asparaginase from *Salinicola acorporae* S4-41

**DOI:** 10.1186/s12934-025-02855-1

**Published:** 2025-11-13

**Authors:** Abd El Raheem El-Shanshoury, Hadeer Helmy Abosamra, Tamer Elsakhawy

**Affiliations:** 1 Bacteriology Unit, Microbiology Section, Botany and Microbiology Department, Faculty of Science, Tanta University, Tanta, 31527 Egypt; 2 Agriculture Microbiology Research Department, Soil, Water and Environment Research Institute (SWERI), Agriculture Research Center (ARC), Sakha Agricultural Research Station, 33717 Kafr El-Sheikh, Giza, Egypt

**Keywords:** L-Asparaginase free from glutaminase, Halotolerant bacteria, *Salinicola acroporae* strainS4-41, Purification, Characterization, Antitumor

## Abstract

**Background:**

The medical community has long searched for a safer and more effective cancer treatment method. Clinically, L-asparaginase derived from bacteria has shown promising results. However, it carries the risk of immunological and hypersensitive reactions due to its interaction with glutaminase. Therefore, finding a new source of glutaminase-free L-asparaginase (ASNase) is important.

**Results:**

In this study, 35 bacterial isolates from 12 saline environmental samples were tested for ASNase, and the most promising bacterium was identified as *Salinicola acroporae* S4-41. This bacterium produces intracellular ASNase, which could treat cancer with fewer side effects. Agitation enhanced production by nearly five-fold compared to static conditions. The enzyme activity peaked at 40 °C and pH 8 after 96 h in a medium containing 100 g L^-1^ of NaCl, 2 g L^-1^ of glucose, and 15 g L^-1^ of L-asparagine. The purified enzyme was 65 kDa in size, with optimal activity in 1 M NaCl, 0.05 M L-asparagine, 0.1% DMSO, urea, and Tween 80 at pH 8 and 40 °C. The enzyme’s kinetics showed a Km of 0.007271 mM and a Vmax of 84.31 U mL⁻¹ min⁻¹. Pure ASNase significantly inhibited the growth of cancer cell lines NB4, MCF-7, and HepG-2. *S. acroporae* produces fewer harmful ASNase enzymes, which could improve cancer treatment. This is the first report of ASNase production from *S. acroporae* S4-41.

**Conclusion:**

*Salinicola acroporae* S4-41 (GenBank accession number SUB12091824; OP521775), a halotolerant bacterium isolated from the rhizosphere soil of cogon grass (*Imperata cylindrica*), was found to produce glutaminase-free L-asparaginase, a significant benefit for lowering treatment-related side effects, making it a promising candidate for safer therapeutic applications. Quantitative analysis revealed an activity of 10.72 U/mL, which increased significantly under optimized physicochemical conditions. Its maximal output (58.76 U/mL) attained at 40 °C and 1.5% L-asparagine, and it also showed notable pH stability at pH 8. The isolate showed improved enzyme output under shaking conditions and adaptability over a wide salinity range (0–25% NaCl). Although activity decreased with ethanol and inhibitors such SDS and EDTA, the isolated enzyme maintained significant stability in the face of heat, pH, and nonionic surfactants (Tween 80, DMSO). These findings demonstrate the enzyme’s surfactant-stable, halotolerant, and thermotolerant characteristics could serve as a safer alternative to currently used bacterial asparaginases by reducing adverse immunological reactions, highlighting its potential for use in pharmaceutical and biotechnological applications. To confirm its medicinal potential and increase its industrial utility, more research is necessary, especially in vivo assessments.

## Key points

- A halotolerant bacterium produces a novel L-asparaginase devoid of glutaminase.
- The enzyme produced under stressful conditions is effective against cancer cell lines.
- Further research required to offer higher amounts of fewer side effects in therapy.

## Introduction

L-asparaginase (ASNase, EC3.5.1.1) is an L-asparagine (L-ASN) amidohydrolase enzyme, which catalyzes non-essential L-ASN into L-aspartic acid (L-ASP) and ammonia [1]. This enzyme is produced by a variety of living organisms, including some plants [2], mammals [3], and microorganisms [4–11]. Bacterial L-asparaginases are generally classified into two main types: cytoplasmic (Type I) and periplasmic (Type II) isoforms [12]. L-asparaginase’s use is limited by its inadequate glutaminase activity, which results from overlapping substrate recognition and conserved catalytic motifs, particularly within the active site triad [13, 14].

The enzyme’s ability to recognize both amino acids allows it to hydrolyze glutamine as well, producing side effects such as neurological complications (seizures, encephalopathy) due to disrupted neurotransmitter synthesis, immunosuppression from reduced lymphocyte proliferation and function, metabolic toxicity impacting the liver and pancreas, and therapeutic limitations with dose reductions (30–40%) and discontinuations (15–20%) [15, 16].

Halophilic bacteria are organisms that survive in extremely salty conditions, and their enzymes are naturally adapted to function at high ionic strengths. These enzymes frequently show strict substrate selectivity, allowing bacteria to efficiently undertake specialized nitrogen metabolism even in nutrient-poor, hypersaline conditions [17–19]. This distinguishes them as potentially effective biocatalyst suppliers.

Cultivating halophilic bacteria at scale is physically challenging because they require specific salt concentrations. Furthermore, little is known about the enzymes’ structural and functional interaction, making it difficult to adapt or modify them for specific purposes. Another issue is that when these enzymes are expressed in common research laboratories or industrial organisms (heterologous hosts such as *E. coli*), their activity and stability are often unpredictable [20]. L-asparaginase is a biomedically significant enzyme used to treat acute lymphoblastic leukemia (ALL). However, current L-asparaginase formulations frequently contain glutaminase activity, which has serious side effects, including harm to healthy tissues. Discovering or development of glutaminase-free L-asparaginase variants from halophilic bacteria would represent a substantial therapeutic advance. Such changes may reduce treatment-related toxicity while maintaining the enzyme’s anticancer activity, so improving the safety and efficacy of ALL treatment [4, 15, 21–23].

Commercial ASNases currently used in the pharmaceutical and food industries are produced by microorganisms, such as bacteria and fungi [4–11]. One possible method for treating cancer is amino acid depletion therapy. It takes advantage of the variations in metabolic pathways between malignant and healthy cells. Some microbial enzymes cause the death of cancer cells via elimination of vital amino acids. The FDA has authorized the enzyme L-asparaginase for the treatment of acute lymphoblastic leukemia [4, 5, 9, 12, 21, 22]. The enzyme L-asparaginase, which has numerous applications, mainly catalyzes the breakdown of the amino acid asparagine. Cancer cells, particularly in lymphoblastic leukemia (ALL), depend heavily on external supplies of L-asparagine. Due to reduced levels of asparagine synthetase in these malignant cells, their capacity to produce L-asparagine is lower than in normal cells. The enzyme limits asparagine availability to cancer cells, which is essential for their growth and survival. This results in the cessation of protein production in leukemic cells, leading to cell cycle arrest, apoptosis (programmed cell death), and ultimately cell death, perhaps with some side effects. Asparaginase degrades asparagine into aspartic acid and ammonia, lowering the development of acrylamide, a possible carcinogen, during high-temperature food processing [3, 5, 9, 12, 15, 21–24].

In addition to its use in the food and medical industries, L-asparaginase has proven to be an effective biosensor. L-asparaginase biosensors can be used to distinguish between leukemic and normal cells [27]. Furthermore, ASNases have been proposed as a potential molecular target for limiting the pathogenicity of *Mycobacterium tuberculosis* and *Salmonella typhimurium* [25]. However, the application of L-asparaginases is limited due to their low activity, poor thermal stability, and adverse reactions during treatment, such as immunogenicity, as well as undesirable glutaminase activity [5–10, 18–21, 23], all of which hinder the applications of this enzyme.

Many researchers are interested in discovering new sources of ASNase with improved thermal stability [26, 28–30], excellent properties for disease chemotherapy [3–5, 9, 12,13, 15, 17, 21, 24, 31, 32], and significant potential in the food processing industry [2, 6–8, 10, 18, 20, 25, 26, 28–30]. The purpose of this work was to develop L-asparaginase-producing halophilic bacteria by screening for glutaminase-free activity, establishing optimal production conditions, and assessing their structural, kinetic, and cytotoxic characteristics.

## Materials and methods

### Samples collection

Rhizosphere soil samples from cogon grass (*Imperata cylindrica*) inhabiting saline soil and water samples from hypersaline environments in Egypt were collected. Collection locations include Wadi El natron, Borg El-Arab, Upper Egypt, and El Behira. Soil samples were collected from the root zone (0–20 cm depth) and placed in pre-sterilized plastic bags. Water samples were obtained from surface layers (0–30 cm depth) using sterile bottles. All samples were immediately transported to the laboratory iceboxes (4 °C).

### Isolation of bacterial isolates

Soil or water samples were inoculated into a saline nutrient broth medium containing 15% (w/v) sodium chloride. The medium was adjusted to pH 7.0 using 0.1 M HCl or NaOH and distributed in 250 ml Erlenmeyer flasks, where each flask received 100 ml. The medium was then autoclaved at 121 °C for 20 min. After cooling to room temperature, 10% (v/v) of each sample (soil or water) was added to the medium and incubated at 30 °C for 72 h in a rotary shaker at 150 rpm. Following incubation, cultures were streaked onto nutrient agar plates with identical NaCl concentration (15% w/v) and incubated at 30 °C for 72 h. After repeated streaking, pure colonies were obtained and then maintained on agar slants of the same medium. In total, 35 morphologically distinct bacterial isolates were selected for further investigation.

### Screening of bacterial isolates for L-asparaginase production

The L-asparaginase production potential of selected isolates was evaluated using a modified rapid plate assay based on Gulati et al. [33]. The assay medium contained (per liter): 6 g Na₂HPO₄·2 H₂O, 3 g KH₂PO₄, 150 g NaCl, 5 g L-asparagine (sole nitrogen source), 0.5 g MgSO₄·7 H₂O, 0.15 g CaCl₂·2 H₂O, 2 g glucose, 20 g agar, and 0.3 mL of 2.5% phenol red solution. For control, NaNO₃ replaced L-asparagine as the nitrogen source. Forty-eight-hour-old broth cultures of each isolate were spotted (50 µL aliquots) onto the agar plates and incubated at 30 °C for 72 h. L-Asparaginase production was indicated by:

1. Phenol Red assay: Formation of a pink halo zone around bacterial growth due to pH shift from asparagine hydrolysis and the release of ammonia.
2. Bromothymol Blue (BTB) confirmation: Appearance of a blue halo zone under identical conditions.

For semi-quantitative analysis, the zone index was calculated as zone index =$\:\:\:\frac{\text{D}\text{i}\text{a}\text{m}\text{e}\text{t}\text{e}\text{r}\:\text{o}\text{f}\:\text{h}\text{y}\text{d}\text{r}\text{o}\text{l}\text{y}\text{s}\text{i}\text{s}\:\text{z}\text{o}\text{n}\text{e}\:\left(\text{m}\text{m}\right)}{\text{D}\text{i}\text{a}\text{m}\text{e}\text{t}\text{e}\text{r}\:\text{o}\text{f}\:\text{b}\text{a}\text{c}\text{t}\text{e}\text{r}\text{i}\text{a}\text{l}\:\text{c}\text{o}\text{l}\text{o}\text{n}\text{y}}$ A larger zone index correlated with higher L-asparaginase activity.

### Qualitative and quantitative checks for L-asparaginase-free glutaminase-producing bacteria

The L-asparaginase-positive isolates were further screened for glutaminase activity by replacing L-asparagine with L-glutamine (5 g/L) in the medium while maintaining all other components and conditions (30 °C, 72 h incubation). Potential glutaminase-free candidates were identified as isolates showing strong pink halos in the L-asparaginase assay but no color change in the glutaminase test. Zone indices were calculated for both assays to quantitatively compare activities. Selected isolates (free of glutaminase activity) were preserved as saline nutrient agar slants at 4 °C for short-term use and in 20% glycerol stocks at -80 °C for long-term storage.

### Cultivation of selected isolates and enzyme production

Based on preliminary screening results, the most promising L-asparaginase-free glutaminase-producing isolate was selected to complete the study. Cultivation was initiated in 250 mL Erlenmeyer flasks containing 50 mL of broth medium used in the screening process (phenol red-free). A single colony inoculum was incubated at 30 °C with shaking (150 rpm) for 72 h. The resulting culture was then diluted with the fresh medium to achieve an optical density of 1.0 at 600 nm (OD600), which was used as the standardized inoculum for subsequent experiments. For enzyme production, 250 mL flasks containing broth medium were inoculated with 2% (v/v) of this cell suspension and incubated under the same conditions (30 °C, 150 rpm, 72 h).

### Determination of enzyme localization

Following 72 h of cultivation in the pre-mentioned broth medium, the culture was centrifuged (6,000 rpm, 20 min, 4 °C) in the ICE DPR-6000 centrifuge. The supernatant was directly assayed for L-asparaginase activity, while the cell pellet was resuspended in 50 mM Tris-HCl buffer (pH 8.0) containing 150 g/L NaCl to maintain osmotic stability. Intracellular enzyme extraction was achieved through sonication on ice (5 cycles of 30 s pulses with 15 s cooling intervals between cycles), followed by centrifugation to remove cell debris. The resulting cell-free lysate was then analyzed for intracellular L-asparaginase activity. Parallel assay of both fractions indicates the enzyme’s primary localization, whether secreted extracellularly or retained intracellularly.

### Enzyme activity assay

L-Asparaginase activity was quantified by measuring ammonia release via the Nesslerization method [34]. For intracellular enzyme analysis, bacterial cultures were centrifuged (6,000 rpm, 20 min, 4 °C), then pellets were washed twice with phosphate buffer (pH 8) before resuspension in the same buffer. Cell disruption was achieved by sonication (5 cycles of 30 s pulses with 15 s cooling intervals on ice), followed by centrifugation to obtain a clear lysate. The reaction mixture contained 0.5 mL of 0.04 M substrate (L-asparagine or L-glutamine), 0.05 mL phosphate buffer (pH 8), and 0.5 mL enzyme solution (crude supernatant or lysate), incubated at 37 °C for 30 min. Reactions were terminated with 0.5 mL of 1.5 M TCA and centrifuged (6,000 rpm, 20 min, 4 °C). For Nesslerization, 0.1 mL supernatant was mixed with 3.7 mL distilled water and 0.2 mL Nessler’s reagent, incubated at 20 °C for 20 min, and absorbance was measured at 450 nm in a Jenway 6705 UV/Vis Spectrophotometer.

Enzyme activity (IU/mL) was calculated using an ammonium sulfate standard curve, where 1 IU equals 1 µmol ammonia released/min/mL under assay conditions [35]. The formula accounted for dilution factors.

Activity = (µmol NH₃) × (2.5/0.1) × (1/30) × (1/0.5), where 2.5 = total reaction volume (mL), 0.1 = sampled volume (mL), 30 = incubation time (min), and 0.5 = enzyme volume (mL).

### Identification of the most potent bacterium

Molecular identification of the most potent bacterium based on 16 S rDNA.

#### DNA extraction and 16 S rDNA PCR amplification

Genomic DNA was extracted from bacterial cultures using the GeneJet Genomic DNA Purification Kit (Fermentas, USA) following the manufacturer’s instructions. The 16 S rRNA gene was amplified in a total reaction volume of 25 µL containing: 18 µL nuclease-free water, 1 µL template DNA, 2 µL forward primer (5′-AGA GTT TGA TCC TGG CTC AG-3′, 10 µM), 2 µL reverse primer (5′-GGT TAC CTT GTT ACG ACT T-3′, 10 µM), and 2× Hot Start Maxima^®^ PCR Master Mix (Fermentas, USA) at the manufacturer’s recommended concentration. PCR reactions were carried out in a thermal cycler under the following conditions: initial denaturation at 95 °C for 10 min, followed by 30 cycles of denaturation at 95 °C for 30 s, annealing at 65 °C for 1 min, and extension at 72 °C for 1 min. A final extension step was performed at 72 °C for 10 min. PCR products were verified by agarose gel electrophoresis.

#### Determination of the topology of the 16 S rRNA sequencing tree

PCR products were verified by electrophoresis on 1% (w/v) agarose gels before sequencing using an ABI 3730xl DNA sequencer (GATC Biotech, Germany). The obtained sequences were assembled and edited using BioEdit version 5.0.9 software to generate a high-quality contiguous sequence. Multiple sequence alignment was performed using the RDP Sequence Aligner from the Ribosomal Database Project. For phylogenetic analysis, reference sequences were retrieved from the RDP database and compared with our isolate using NCBI BLAST to identify closely related bacterial strains.

Phylogenetic reconstruction was conducted using the neighbor-joining method implemented in MEGA X software (version 10.2.6). The evolutionary distances were computed using the Kimura 2-parameter model with gamma-distributed rate variation among sites (shape parameter = 0.5). The robustness of the phylogenetic tree topology was assessed by bootstrap analysis with 1000 replicates. The final 16 S rRNA gene sequence was deposited in the GenBank database.

#### Optimization of L-asparaginase production conditions

To maximize L-asparaginase yield, culture conditions were optimized using a one-factor-at-a-time approach. The study evaluated incubation periods (1–7 days), temperature (25–45 °C in 5 °C increments), initial pH (5.0–9.0), and aeration (static vs. shaking at 150 rpm). Nutritional parameters included various carbon sources (0.2% w/v glucose, sucrose, mannitol, fructose, starch, sodium acetate, or aspartame) and nitrogen sources (beef extract, peptone, yeast extract, asparagine, urea, ammonium chloride, ammonium acetate, or sodium nitrate), with nitrogen concentrations tested from 0.2% to 2% w/v. On the other hand, salinity effects were conducted using NaCl (0–25%). For each trial, L-asparaginase activity was measured via the Nesslerization assay, while bacterial growth was measured as turbidity at 600 nm (OD600).

### Enzyme purification

#### Ammonium sulfate precipitation

The crude enzyme extract was purified at 4 °C following established methodologies [36, 37]. Initial purification involved filtration with a 0.22 μm filter membrane to remove particulate impurities, followed by stepwise ammoniumsulfate precipitation (0–80% saturation). The solution was incubated overnight at 4 °C. Precipitated proteins were recovered by centrifugation (6000 rpm, 20 min, 4 °C) and then resuspended in 50 mM phosphate buffer (pH 8.0). The resolubilized fraction was dialyzed (12–14 kDa cutoff membrane) against distilled water for 24 h to remove residual salts.

#### Gel filtration chromatography

Dialyzed samples were further purified using a Sephadex G-100 column (45 × 1.5 cm) pre-equilibrated with 50 mM phosphate buffer (pH 8.0). A 5 mg protein aliquot was loaded and eluted at 20 °C with the same buffer (flow rate: 1 mL/min), collecting 3 mL fractions. Protein elution profiles were monitored at 280 nm, and L-asparaginase activity was assayed in all fractions [34].

#### Pooling and storage

Fractions exhibiting peak enzyme activity were pooled and dialyzed against 50 mM phosphate buffer (pH 8.0) to remove low-molecular-weight contaminants. The purified enzyme was stored at 4 °C for subsequent characterization. Protein concentration and specific activity were determined at each purification stage to evaluate yield and fold purification.

### Protein determination

Protein concentration was determined using the Lowry method [38] with bovine serum albumin as the standard and a calibration curve ranging from 0 to 100 mg/L.

#### Calculation of the molecular weight

SDS-PAGE was used to determine the molecular weight of partially purified L-asparaginase [39]. The silver stain was first used to stain the gel, according to [40], then Coomassie brilliant blue.

#### Characterization of enzyme activity

##### Biochemical characterization of purified L-asparaginase

1. Optimal activity conditions.
  The purified enzyme was characterized under varying physicochemical conditions to determine its functional parameters. The optimal pH was assessed across a range of 5.0–10.0 using 50 mM buffers (acetate for pH 5.0–6.0, phosphate for pH 7.0–8.0, and glycine-NaOH for pH 9.0–10.0). Temperature stability was evaluated from 30 to 60 °C, and halotolerance was tested with NaCl concentrations (0–1.25 M). All assays used a fixed enzyme concentration (4.85 mg/mL) in 50 mM phosphate buffer (pH 8.0).
2. Chemical modulators.
  The enzyme’s sensitivity to surfactants (0.1% Tween-80, 1% SDS, 1 M urea, 5 mM EDTA), organic solvents (0.1% DMSO, 50% ethanol), and metal ions (1 mM Fe²⁺, Cu²⁺, Co²⁺, Zn²⁺, Mg²⁺, Ba²⁺) was analyzed. Residual activity was measured after 1 h incubation at 37 °C relative to untreated controls.

3. Stability profile.
  Storage stability:Activity retention was monitored in 50 mM phosphate buffer (pH 8.0) at 4 °C and − 20 °C over 30 days.
  Thermal stability: The stability of the enzyme at 37 °C was assessed by measuring its residual activity for 2 h. Aliquots were withdrawn and immediately cooled on ice to stop further thermal inactivation. The residual enzymatic activity was measured under standard assay conditions and expressed as a percentage of the initial activity.
4. Kinetic analysis.
  Michaelis-Menten parameters were derived using L-asparagine (0.01–0.1 M) in 50 mM phosphate buffer (pH 8.0). Initial reaction rates were plotted via.
  Lineweaver-Burk analysis: Linear regression of double-reciprocal plots.
  Non-linear regression: Direct fit to the Michaelis-Menten equation (GraphPad Prism, La Jolla, CA).

### Evaluation of human serum effects on L-asparaginase activity and stability

To assess the impact of human serum components on L-asparaginase functionality, the purified enzyme was incubated in human serum (1:1 v/v) at 37 °C for 30 min, followed by immediate measurement of residual activity using the standard Nesslerization assay. This tested both the enzyme’s susceptibility to serum-borne inhibitors and its compatibility with physiological conditions.

For thermal stability analysis, L-asparaginase was incubated in human serum at 37 °C, with aliquots withdrawn hourly over 4 h. Residual activity was quantified at each time point to determine the enzyme’s half-life (t₁/₂) under simulated physiological conditions. Control reactions (enzyme in buffer alone) were run in parallel to distinguish serum-specific effects from thermal denaturation.

### The in vitro cytotoxic tests

#### Cell culture and maintenance

Dulbecco’s modified Eagle’s medium (DMEM) is used to cultivate human hepatoma HepG2, breast cancer cells MCF7, human acute promyelocytic leukemia (NB4), and healthy Madin Darby Canine Kidney MDCK cell lines. All media were supplemented with fetal bovine serum (FBS) and 1% penicillin/streptomycin (Seralab, UK). The cells were raised in 5% CO₂ that was humidified and kept at 37 °C to sustain growth. All investigations related to tissue culture have followed the established procedures.

#### MTT test evaluation of cell proliferation

The 3-[4,5-methylthiazol-2-yl]-2,5-diphenyl-tetrazolium bromide (MTT) test, as previously described by [41, 42], was used to determine the amounts of live HepG2, NB4, and MCF7 cells after treatment with different enzyme doses. In conclusion, a method based on trypan blue dye counts determines the viability of the cells. The cancer cells (10^4^/well) were seeded on a 96-well plate and allowed to be attached overnight. The following day, the entire medium was replaced with a fresh one, and various enzyme concentrations were measured (1, 10, 100, and 1000 µg /mL) in every cell line. The cells were then left to continue growing for an additional 24 h. Each well received 10 µl of the MTT (5 mg/ml) solution four hours before the end of the incubation period. Each well received 100 µl of dimethyl sulfoxide (DMSO) following the incubation period (DMSO), which was then given time to dissolve the formazan crystals for 20 min. The dye dissolution was then checked for uniformity by shaking the 96-well plates for 5 min. After the reaction, a Bio-Tek microplate reader was used to measure the color development at 490 nm.

#### Apoptotic assay

We performed apoptosis studies in cancer cells with acute promyelocytic leukemia using well-established methods [41, 43]. 1.0 × 106 cells/flask were inoculated for 24 h, and then NB4 cells were exposed to the enzyme’s IC50 dose (513 µg /ml). Cells were harvested with trypsin after a 48-hour incubation period and then fixed using the Annexin V-FITC Detection Kit’s directions (Catalog #: K101-25, Bio Vision). The proportion of apoptotic cells was then ascertained using flow cytometry. Apoptosis studies using proven methods were performed on cancer cells with the characteristics of acute promyelocytic leukemia.

### Statistical analysis

The analysis of variance (ANOVA) was performed on the measured data using a fully randomized one-way design in triplicate; mean values and standard deviation were estimated. Using the Costas program, Duncan’s test has been used to assess significant differences between treatments at a 5% probability level.

## Results

### Isolation of bacteria and primary screening

**Fig. 1:**
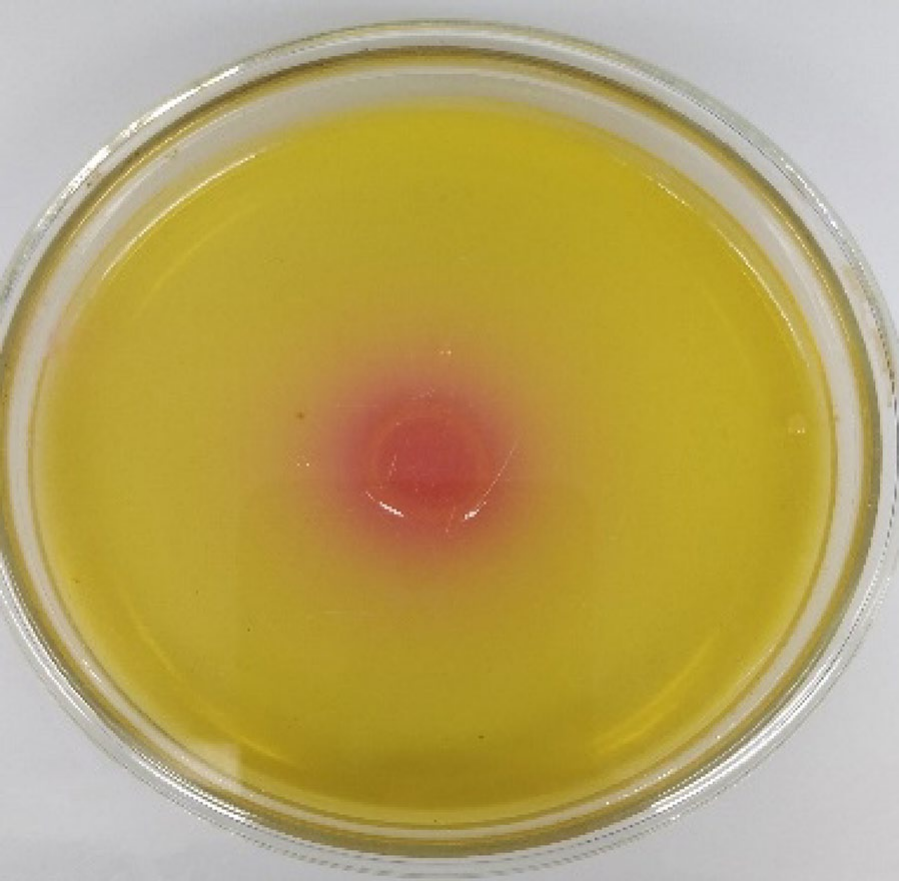
*Salinicola acroporae* grown on agar medium containing L-asparagine as a sole nitrogen source and phenol red as indicator, the pink halo around the colony indicates asparaginase production and asparagine hydrolysis releasing ammonia and raising pH

From 12 marine water/soil samples, 35 bacterial strains were isolated. Initial screening using phenol red indicator (Fig. 1) revealed that 22 isolates exhibited both asparaginase and glutaminase activity, while only one bacterial isolate (from rhizosphere soil samples of cogon grass (*Imperata cylindrica*)) showed asparaginase-free glutaminase activity. This glutaminase-free isolate was confirmed through secondary screening with bromothymol blue (BTB), which produced analogous results (Table 1).

### Semiquantitative and quantitative analysis

The selected isolate produced intracellular L-asparaginase, where no activity was detected in the supernatant. Semi-quantitative assays yielded zone indices of 9.17 (phenol red) and 9.29 (BTB). Crucially, the isolate failed to grow on medium containing glutamine as a sole carbon source, confirming the absence of glutaminase activity. Quantitative analysis demonstrated an activity of 10.72 U/mL with no detectable glutaminase activity (Table 1).

**Table 1:** Semiquantitative detection of glutaminase-deficient L-asparaginase-producing isolate

Indicators	Phenol Red Indicator		Bromothymol Blue Indicator	
Amino acid	Asparagine	Glutamine	Asparagine	Glutamine
Zone index	9.17	0	9.29	0

### Molecular identification of the most effective bacterium

**Fig. 2:**
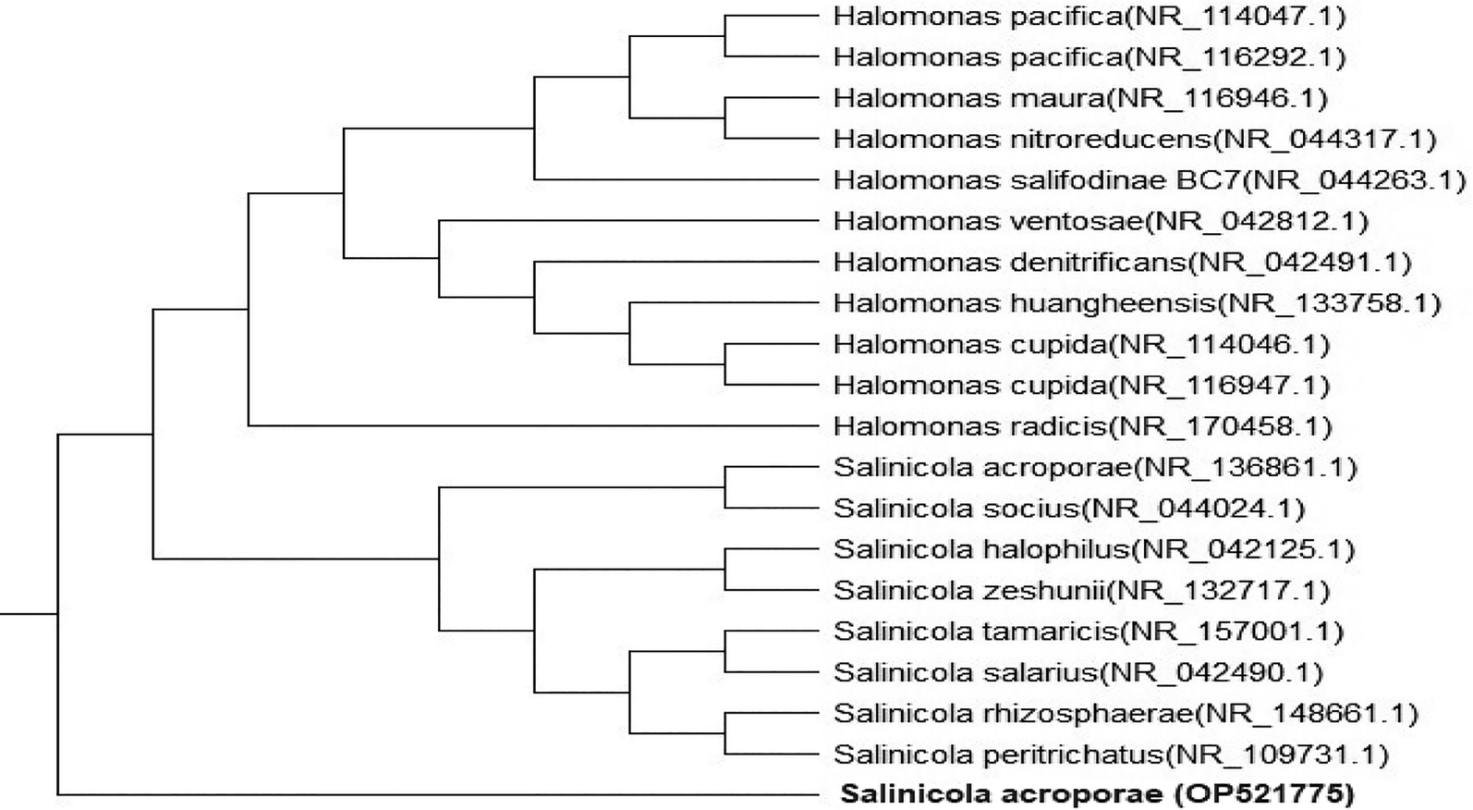
Phylogenetic analysis of *Salinicola acroporae* and related taxa based on 16 S rRNA gene sequences

The marine bacterium that did not grow on glutamine medium was selected as a possible new candidate for L-asparaginase production based on screening results from earlier studies. Considering the outcomes of the NCBI GenBank BLAST search and the evolutionary tree (Fig. 2), it was discovered that strain S4-41 of *Salinicola acroporae* was the closest match to the isolate that produced L-asparaginase. The distance matrix and nucleotide alignment displayed a high similarity value of 90.68%, consistent with previous results obtained via the BIOLOG system. The identification of the strain was confirmed, and the GenBank database received it under the accession number SUB12091824 Salinicola OP521775.

### Optimization of L-asparaginase production

The results of changing the growing conditions to improve L-asparaginase production are shown in Fig. 2.

#### Culture condition optimization

The production of L-asparaginase by the selected halotolerant isolate was optimized under various physicochemical conditions. Maximum enzyme activity (13.09 U/mL) was observed after 4 days of incubation, with a noticeable decline to 6.90 U/mL by day 7, suggesting a depletion of essential nutrients or a response to the accumulation of catabolites. Agitation significantly enhanced enzyme yield, where cultures incubated under shaking conditions (150 rpm) exhibited a 4.8-fold increase in activity compared to static incubation (24.69 vs. 5.69 U/mL). pH played a critical role in modulating enzyme production. The optimal activity was recorded at pH 8 (15.49 U/mL), while deviations toward acidic or more alkaline ranges led to significant reductions in enzyme production. The isolate demonstrated remarkable halophilicity, with optimum L-asparaginase production at 10% NaCl. The enzyme retained 40–60% of its activity across a broad salinity range (0–25% NaCl), underscoring its adaptability to variable salinity concentrations.

#### Nutritional requirements

**Fig. 3:**
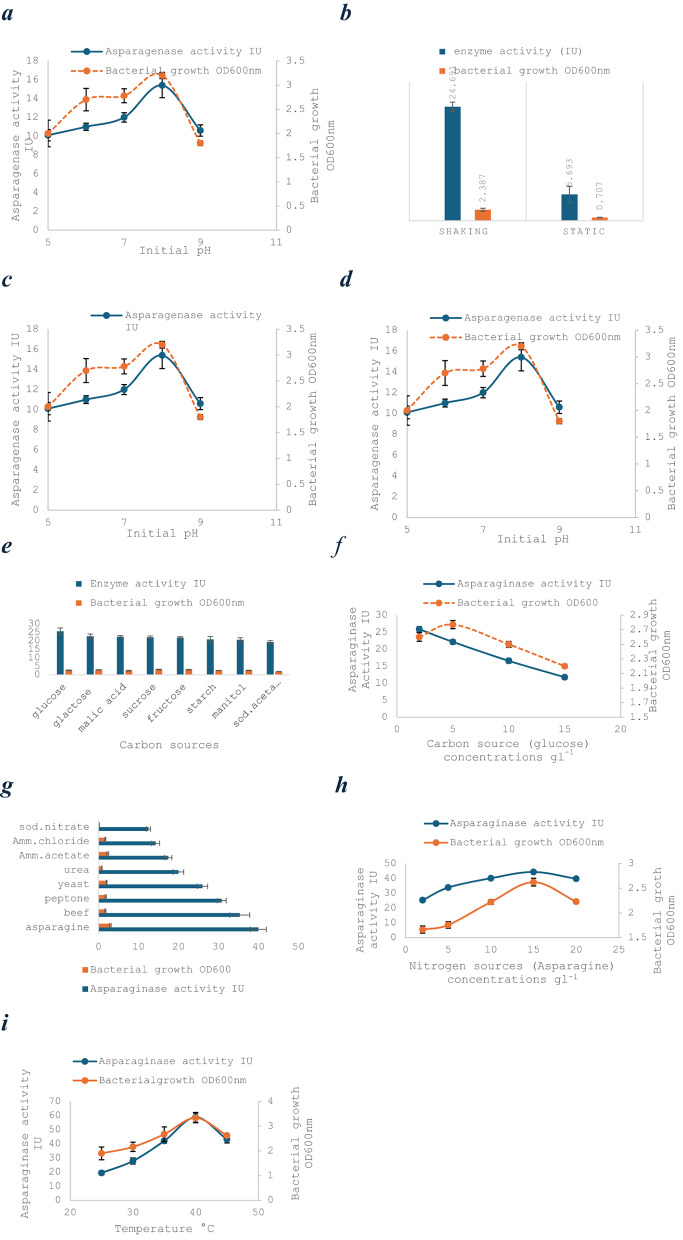
Optimization of culture conditions for *Salinicola acroporae* growth (bars) and L-asparaginase production (lines). Data represent mean ± SD of three biological replicates. **a** Incubation time (24–168 h); **b** Aeration conditions (static vs. 150 rpm); **c** Initial pH (5.0–9.0); **d** NaCl concentration (0–25% w/v); **e** Carbon sources (0.2% w/v); **f** Glucose concentration (0.1–2.5% w/v); **g** Nitrogen sources (0.2% w/v); **h** L-asparagine concentration (0.2-2.0% w/v); **i** Temperature (25–45 °C). *p < 0.05, **p < 0.01, ***p < 0.001 vs. optimal condition (one-way ANOVA with Tukey’s test)

Among various tested carbon sources, glucose yielded the highest L-asparaginase activity (25.60 U/mL), significantly outperforming other carbon sources. Nitrogen source optimization revealed that L-asparagine at 1.5% (w/v) supported the highest enzyme production (39.98 U/mL), surpassing alternative organic and inorganic nitrogen sources by two- to five-fold. Temperature was another key determinant, with optimal production (58.76 U/mL) achieved at 40 °C. This represented a three-fold increase relative to production observed at 25 °C, reflecting the thermotolerant nature of the enzyme system (Fig. 3a, b, c, d, e, f, g).

#### Optimization of carbon source concentration

The influence of glucose concentration (0.2–1.5% w/v) on L-asparaginase production was evaluated. Maximum enzyme activity (25.87 U/mL) occurred at 0.2% glucose, with progressively lower yields at higher concentrations (11.76 U/mL at 1.5%) (Fig. 3f). Notably, biomass production remained statistically unchanged across all tested concentrations, which indicates that carbon source levels specifically modulated enzyme synthesis rather than growth. This inverse relationship between glucose concentration and asparaginase productivity suggests potential catabolite repression at elevated sugar levels.

#### Optimization of nitrogen source concentration

The effect of L-asparagine concentration (0.2–2.0% w/v) on *Salinicola acroporae* was investigated. Maximum L-asparaginase activity (44.29 U/mL) and bacterial growth (OD = 2.62) were achieved at 1.5% L-asparagine (Fig. 3h). Enzyme production and growth decreased significantly at both lower and higher concentrations (ANOVA, *p* ≤ 0.05), demonstrating strict concentration dependence. These results indicate that 1.5% L-asparagine represents the optimal balance between nitrogen availability and potential metabolic inhibition for both enzyme production and biomass accumulation in this halophilic strain.

#### Purification and characterization

**Fig. 4:**
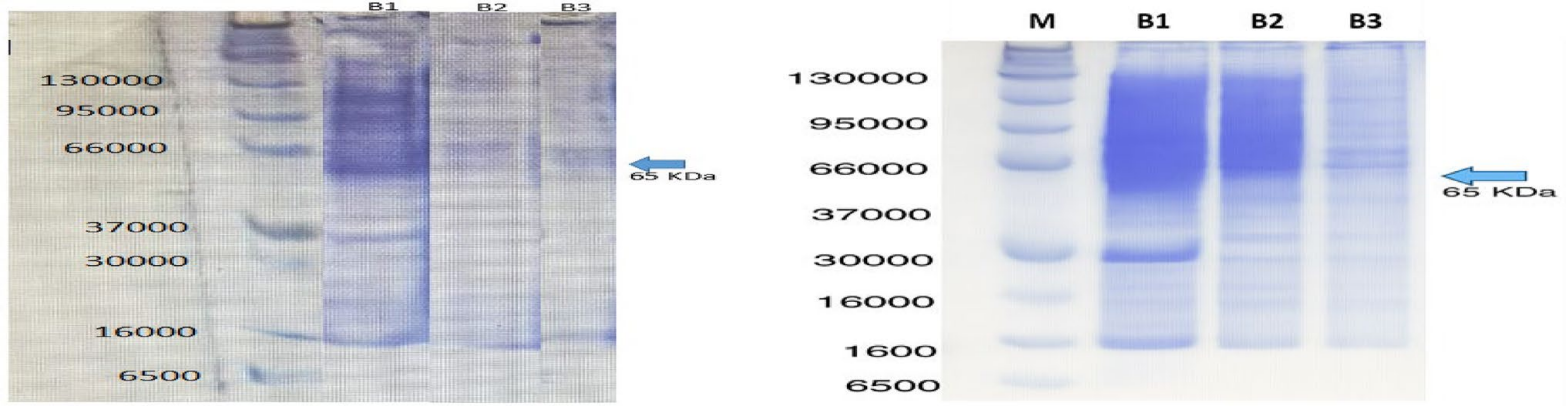
SDS-PAGE (12%) analysis of L-asparaginase purification from *Salinicola acroporae*. Lane M: Precision Plus Protein™ Standards (kDa); Lane B1: Crude enzyme extract; Lane B2: 80% ammonium sulfate precipitate; Lane B3: Gel filtration chromatography fraction showing purified L-asparaginase (~ 65 kDa). The gel image was digitally contrast-enhanced solely to improve band visibility; no alterations were made to the experimental data

The purification of L-asparaginase resulted in an 11.6-fold increase in specific activity, with an overall recovery of 7%. Initial ammonium sulfate precipitation achieved 6.3-fold purification and 40% yield. Subsequent gel filtration chromatography using Sephadex G-100 yielded a final specific activity of 138 U/mg (Table 2). SDS-PAGE analysis confirmed the homogeneity of the purified enzyme, revealing a single prominent band corresponding to a molecular mass of approximately 65 kDa (Fig. 4).

**Table 2:** Purification scheme for L-asparaginase from *Salinicolaacroporae*

Purification stage	Volume (mL)	Enzyme activity (IU/mL)	Protein (mg/mL)	Total activity (IU)	Total protein (mg)	Specific activity (IU/mg)	Yield (%)	Purification fold
Crude extract	150	58.02	4.85	8703	727.5	11.96	100	1.0
Ammonium sulfate precipitation	20	174.48	2.32	3489.6	46.4	75.21	40	6.3
Gel filtration (Sephadex G-100)	3	211.17	1.52	633.5	4.56	138.93	7	11.6

#### L-asparaginase characterization

**Fig. 5:**
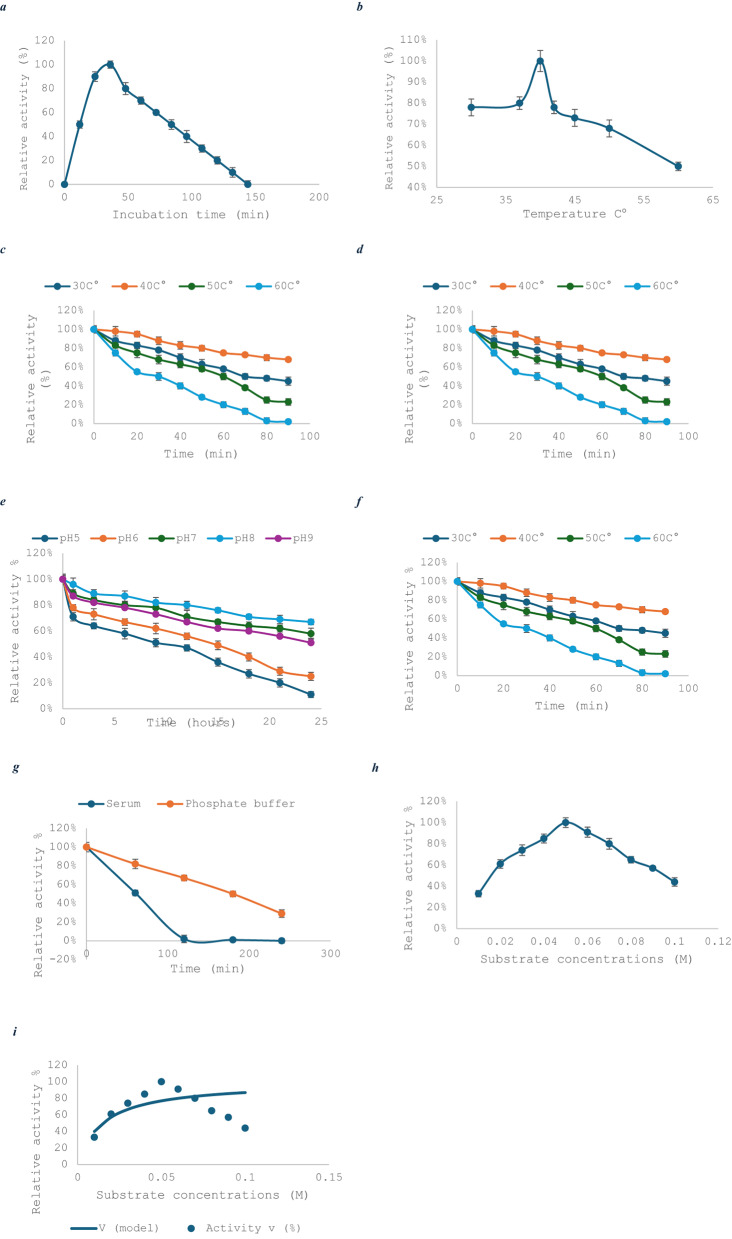
Biochemical characterization of purified L-asparaginase. **a** Optimal incubation time; **b** Temperature optimum; **c** Thermal stability at 40 °C; **d** pH optimum; **e** pH stability at pH 8.0; **f** NaCl tolerance; **g** Stability in phosphate buffer vs. human serum at 37 °C; **h** Substrate saturation curve; **i** Michaelis-Menten kinetics (Km = 0.0073 M, Vmax = 84.31 U ml⁻¹ min⁻¹). All data points represent mean ± SD (*n* = 3). Dashed lines in (**c**, **e**, **g**) indicate 50% residual activity

The effect of different incubation times on the purified L-asparaginase activity was illustrated in Fig. 5a for *S. acroporae*, which revealed that the highest L-asparaginase activity was recorded at 30 min of incubation, where the relative activity percentage was 100%. Additionally, the results determined how the temperature affected the stability and activity of L-asparaginase. Our findings showed that L-asparaginase activity increased as the temperature rose, reaching a maximum of 40 °C. Even though the activities were significantly impacted by 80 °C, losing roughly 95% of their total activity, the enzyme activity decreased by about 22% at 30 °C (Fig. 5b). L-asparaginase and its thermal sensitivity are demonstrated in Fig. 5c. At 40 °C, purified L-asparaginase is the most stable and still has more than 75% of its activity. Following the effects of pH on the enzyme’s activities, L-asparaginase exhibits its maximum activity at pH 8. At pH 9.0 and pH 7.0, the enzymes lost about 87% and 78% of their cumulative activity, respectively (Fig. 5d). L-asparaginase recovered with high pH stability, peaking at pH 8, retains 67% of its activity even after incubation for 24 h (Fig. 5e).

According to studies on the effects of surfactants, organic solvents, and enzyme inhibitors on enzyme activity, purified L-asparaginase exhibited variable stability depending on the compound tested. The enzyme showed a significant increase in activity in the presence of urea, reaching 556% of the control value (*p* < 0.001). In contrast, EDTA markedly reduced activity to 76% of control levels (*p* < 0.05). Among surfactants, Tween 80 enhanced enzyme stability, yielding a modest but significant increase to 109% (*p* < 0.05), whereas SDS caused a reduction of approximately 24% compared with the control (*p* < 0.05). Organic solvents exerted inhibitory effects, with 50% ethanol decreasing enzyme activity to 78% and DMSO reducing it to 104%, both statistically significant compared with the control (*p* < 0.05). These results confirm that while certain compounds can improve enzyme stability, most solvents and inhibitors negatively affect L-asparaginase activity (Table 3).

**Table 3:** Impact of surfactants, solvents, and enzyme inhibitors on L-asparaginase activity from *Salinicola acroporae*

Compound	Activity (U) (mean ± SE)	Relative activity (%)	*p*-value vs. Control
Control	211.2 ± 5.3	100%	–
Urea	1174.5 ± 12.7	556%	< 0.001
EDTA	160.7 ± 4.6	76%	< 0.05
Tween 80	229.5 ± 6.2	109%	< 0.05
Ethanol 50%	165.3 ± 5.1	78%	< 0.05
SDS	160.7 ± 3.9	76%	< 0.05
DMSO	220.3 ± 4.8	104%	< 0.05

Data are expressed as mean ± SE (n = 3)

Statistical significance was analyzed using one-way ANOVA followed by post hoc comparisons with the control

Additionally, the effect of various metal ions on L-asparaginase activity was evaluated to better understand the enzyme’s catalytic requirements and potential cofactors. Among the tested ions, Na⁺ (NaCl) exerted the most pronounced stimulatory effect, enhancing enzyme activity to 139% of the control (*p* < 0.001). Moderate activation was also observed in the presence of Mg²⁺ (107%), Br²⁺ (102%), and Co²⁺ (105%), indicating that these cations may play a supportive role in stabilizing the enzyme’s structure or facilitating substrate binding. In contrast, Fe²⁺ and Cu²⁺ reduced activity to 91% and 93%, respectively, while Zn²⁺ exhibited the strongest inhibitory effect, lowering activity to 81% of the control (*p* < 0.01). These findings suggest that while certain ions enhance enzyme stability and function, others particularly Zn²⁺ may interfere with the active site or alter protein conformation, leading to reduced catalytic efficiency (Table 4).

**Table 4:** Metal cations impact on purified L-asparaginase activity from *Salinicola acroporae*

Metal ion (mM)	Activity (U) (mean ± SE)	Relative activity (%)	*p*-value vs. Control
Control	197.4 ± 4.8	100%	–
Cu²⁺	183.7 ± 5.1	93%	< 0.05
Co²⁺	206.6 ± 4.3	105%	ns
Fe²⁺	179.1 ± 6.0	91%	< 0.05
Br²⁺	202.0 ± 5.4	102%	ns
Mg²⁺	211.2 ± 6.2	107%	< 0.05
Zn²⁺	160.7 ± 5.5	81%	< 0.01
Na⁺	274.4 ± 7.2	139%	< 0.001

Data are expressed as mean ± SE (n = 3). Statistical significance was analyzed using one-way ANOVA followed by post hoc comparison with the control

ns = Nonsignificant

Figure 5f depicts how the concentration of NaCl affects L-asparaginase activity. Our results revealed that the activity of L-asparaginase decreased with an increase in the concentration of sodium chloride up to 1.25 M NaCl, where the enzyme activity was 87.37 U/ml. 0.15 M NaCl, or a physiological 0.9% saline solution, has no adverse effects on enzyme activity. Enzyme activity in 1 M NaCl could be decreased by half compared to NaCl-free buffer. At 4 °C, the enzyme’s stability of *S. acroporae* was assessed. The enzyme gradually lost half of its activity in just two weeks and completely lost it over six weeks. The enzyme’s stability at -20 °C was also evaluated, and it lost half of its activity in 9 months (data not shown). At 37 °C and phosphate buffer at 50 mM, the estimated enzyme half-life was 3 h (Fig. 5g). The phosphate buffer within the response mixture was replaced with serum, and a gradual decline in activity was observed. However, after 60 min of incubation at 37 °C with serum, the enzyme became less active with a half-life estimated to be 60 min (Fig. 5g).

Considering the findings in Fig. 5h, enzyme activity increased gradually as substrate concentration increased between 0.001 and 0.1 M, peaking at 0.05 M. The enzymatic activity decreased when the substrate concentration was increased (0.06–0.1 M). The Km and Vmax data for the L-asparaginase enzyme are presented on the Michaelis-Menten plot. Hydrolysis of the bacterial L-asparaginase plot revealed a Km value of 0.007271 mM and a Vmax of 84.31 U mL⁻¹ min⁻¹ (Fig. 5i).

### L-asparaginase as an antitumor

#### Effects of the cytotoxicity of pure L-asparaginase

**Fig. 6:**
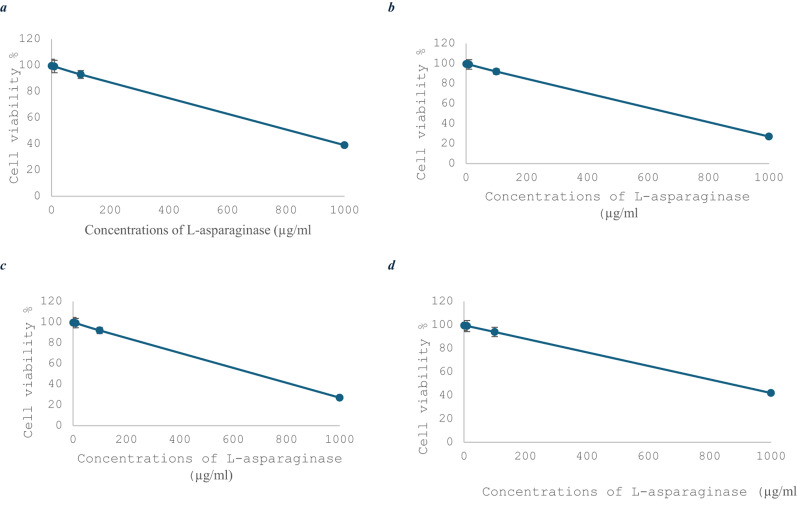
Cytotoxic effect of various concentrations of pure L-asparaginase synthesized by *Salinicola acroporae* on the cell lines MCF-7 (**a**), HepG-2 (**b**), NB4 (**c**), and MDCK (**d**)

The outcome of L-asparaginase was used to investigate the activity of L-asparaginase in cells, and an in vitro cytotoxicity assay was performed on the growth of three distinct lines of cells for cancer cells: HepG-2, NB4, and MCF-7 cells, together with one healthy cell line, MDCK cells. According to the results, L-asparaginase effectively prevents the spread of cancerous cell lines NB4, MCF-7, and HepG-2. Consequently, L-asparaginase is regarded as a potent anticancer agent. In HepG-2, NB4, and MCF-7 cells, the IC50 values of purified L-asparaginase were determined to be 737.4, 513, and 711.5 µg/ml, respectively. The most sensitive cell type for L-asparaginase therapy was NB4 cells (Figs. 6a, b, c, and d).

#### Flow cytometry assay

**Fig. 7:**
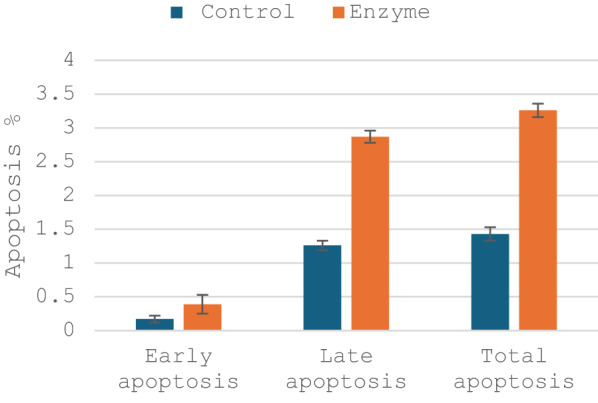
Flow cytometric analysis of apoptosis in NB4 cells treated with L-asparaginase from *Salinicola acroporae*. Bars represent the percentage of early apoptosis, late apoptosis, and total apoptosis in control cells and cells exposed to purified enzyme. Data are presented as mean ± standard error (*n* = 3). Statistical significance was determined using a *t*-test; *p* < 0.05 was considered significant

Dual staining with Annexin V-FITC and propidium iodide (PI) revealed that L-asparaginase from *S. acroporae* induced significant apoptosis in treated cancer cells compared to untreated controls. The enzyme increased early apoptotic cells (Annexin V+/PI-) from 0.17% to 0.39%, while late apoptotic/necrotic cells (Annexin V+/PI+) rose from 1.26% to 2.87% (Fig. 7). This 2.3-fold enhancement in late apoptosis confirms the enzyme’s ability to trigger programmed cell death through its asparagine-depleting mechanism. The Annexin V binding demonstrates phosphatidylserine externalization, a hallmark of apoptosis, while PI incorporation reflects subsequent membrane integrity loss during later stages of cell death.

## Discussion

The screening for glutaminase-free L-asparaginase producers was conducted using the modified Gulati et al. method [33]. This method depends on pH shifts leading to colorimetric changes resulting from ammonia release during asparagine hydrolysis. This assay demonstrated that phenol red transitions to pink while bromothymol blue shifts to blue at pH >7.6. In both cases, the change in color indicates L-asparaginase activity through distinct visual endpoints. The selected isolate exhibited strong enzymatic activity, producing hydrolysis zones measuring 13.57 mm with phenol red and 13.29 mm with bromothymol blue. These zone sizes categorize it as an excellent L-asparaginase producer according to the classification system established by Devi and Ramanjaneyulu [45], where zones of 9–12 mm indicate good producers.

On the other hand, the absence of color change in glutamine-containing media provided clear evidence of the isolate’s lack of glutaminase activity. This finding supports the concept that asparaginase and glutaminase activities are distinct enzymatic functions [44], where, in the case of our isolate, it specifically hydrolyzes the amide bond in L-asparagine to release ammonia without affecting glutamine.

According to the current results, the isolate identified as *Salinicola acroporae* is classified for the first time as a glutaminase-free L-asparaginase producer. The production of enzyme in submerged culture under shaking conditions aligns with previous reports showing that moderate agitation enhances both microbial growth and enzyme production [46, 47]. Specifically, Heinemann and Howard [47] observed similar aeration-dependent growth patterns in *Serratia marcescens*, suggesting this may be a common requirement among L-asparaginase producers. In addition, the current study supports the established role of sodium chloride in promoting the L-asparaginase synthesis in halophilic [48].

The physiological characterization revealed that *S. acroporae* achieved maximal L-asparaginase production (58.76 U/mL) at 40 °C under submerged cultivation (150 rpm). This finding aligns with reports by Amena et al. [36] for *Streptomyces gulbargensis* but contrasts with several other microbial systems: while *Bacillus firmus*, *Streptococcus* spp., and *B. circulans* showed optimal activity at 45 °C [49], other species, including *Erwinia* spp., *Lysinibacillus fusiformis* B27 [50, 51], *Streptomyces albidoflavus*, *B. polymyxa*, and *Paenibacillus spp.* [52] , exhibited peak production at 35 °C. Still other producers, such as *Serratia marcescens* [53] and *Pseudomonas stutzeri* [54, 55], demonstrated temperature optima at 30 °C and 37 °C, respectively.

Our study identified pH 8.0 as optimal for L-asparaginase production, aligning with findings for *Bacillus polymyxa* and *Streptococcus* spp [49]. This alkaline preference appears consistent across several actinobacterial producers, including *Streptomyces albidoflavus* [58] and *S. ginsengisoli* (pH 8.0–9.0) [56, 57]. The recurrent observation of maximal activity at pH 8.0–9.0 among diverse microbial systems suggests this range may be particularly favorable for maintaining both enzyme stability and cellular secretion mechanisms in neutrophilic to alkaliphilic L-asparaginase producers.

The current study demonstrated that glucose served as the optimal carbon source for L-asparaginase production in *S. acroporae.* This results in complete accordance with findings by Prakasham et al. [55] for *Staphylococcus sp.* However, significant microbial variability exists in carbon source preferences. Wakil and Adelegan [49] reported mannitol, sucrose, and maltose as optimal for *Bacillus firmus* and *Streptococcus* spp., while Kenari et al. [59] identified lactose as most effective. Concentration dependence also varied across species, with optimal glucose levels ranging from 10 g L⁻¹ [36, 52] to 20 g L⁻¹ [53, 55], though our results aligned with *Fusarium oxysporum* F-S3, showing peak production at 0.2% (w/w) glucose [60]. This variability likely reflects species-specific metabolic pathways and regulatory mechanisms governing L-asparaginase synthesis.

On the other hand, L-asparagine (15 g L⁻¹) was identified as the optimal nitrogen source for L-asparaginase production in *S. acroporae*, consistent with findings by Nawaz et al. [61] and supporting the common observation that asparagine serves as both substrate and inducer for many microbial L-asparaginases [9, 49, 52, 62, 63]. However, nitrogen source preferences varied significantly among different producers: while peptone was most effective for some systems [57], *Staphylococcus sp.* showed different nutritional requirements [55]. Concentration dependence also exhibited strain-specific patterns, with *S. glbargensis* achieving optimal production at much lower asparagine concentrations (2 g L⁻¹) [36]. The optimal L-asparagine concentration (1.5% w/v) for *S. acroporae* aligns with its dual metabolic role as both a nitrogen source for L-asparaginase production and a carbon source via aspartate catabolism, as demonstrated in other microbial systems [61]. This is supported by the significantly lower glucose requirement (0.2% w/v) compared to conventional producers like *E. coli* (1–2% glucose) [55, 79], suggestingefficient utilization of asparagine-derived carbon through the aspartate-oxaloacetate pathway [85]. Such metabolic flexibility mirrors observations in *Bacillus spp.*, where amino acid catabolism reduces glycolytic demand [63]. The activity decline above 1.5% asparagine may reflect ammonia-mediated inhibition [84] or carbon-nitrogen imbalance, consistent with halophilic metabolic constraints [52].

As for the effect of metal ions on enzyme production, 0.03% Cu²⁺ is the optimal metal ion for L-asparaginase production by *S. acroporae*, aligning with Dumina et al. [64], who reported enhanced enzyme yields (1.71–1.74 IU) with trace metals (Cu²⁺, Zn²⁺, Mn²⁺ at 0.001–0.002%) in H82 medium. The observed metal ion effects likely relate to their roles as enzyme cofactors or regulators of cellular metabolism during asparaginase production [64]. The maximum enzyme production occurred after 96 h of incubation, signifying this duration is the ideal balance between microbial growth and enzyme synthesis kinetics.

L-Asparaginase from *Salinicola acroporae* was purified through 80% ammonium sulfate precipitation [65] followed by Sephadex G-100 gel filtration. SDS-PAGE (12%) revealed a single band at 65 kDa, indicating homogeneity. This molecular weight falls within the wide range reported for microbial L-asparaginases: from 33 to 34 kDa in *Thermus thermophilus* and *Pseudomonas stutzeri* [26, 70] to 80 kDa in *Corynebacterium glutamicum* [66], and up to 140–153 kDa in various *Streptomyces* species [67, 68, 71, 72]. Notably, molecular weights of 85 kDa (*S. gulbargensis*), 112–116 kDa (*S. albidoflavus*) [52], 120 kDa (*S. noursei*), 140 kDa (*Streptomyces* PDK2) [67, 68], and 97.4 kDa (*S. tendae*) [69] have been reported, demonstrating significant variation across bacterial sources.

Following purification from *Pseudomonas aeruginosa* 50,071, SDS-PAGE analysis revealed the L-asparaginase exhibited a molecular mass of 160 kDa [73]. *Salinicola acroporae* L-asparaginase under study demonstrated optimal activity after 30 min of incubation, achieving maximal substrate hydrolysis rates also. The enzyme displayed a temperature optimum of 40 °C, which is particularly relevant for therapeutic applications as it approximates human body temperature. This thermal profile suggests potential stability under physiological conditions.

The purified L-asparaginase from *S. acroporae* demonstrated optimal activity at pH 8.0, aligning with previous reports for similar enzymes [67]. This alkaline preference is particularly relevant for therapeutic applications, as the pH-dependent activity profile correlates with the enzyme’s carcinostatic properties. Our investigations revealed that Mg²⁺ serves as a critical activator, likely through stabilization of the enzyme-substrate complex and maintenance of structural integrity. The enzyme exhibited robust performance in biologically relevant conditions, maintaining full activity in both saline and serum environments. Comparative studies showed superior functionality relative to *E. coli* (40% less active) and *E. chrysanthemi* (80% less active) counterparts [75], with a serum half-life of 60 min at 37 °C intermediate between the more stable *Enterobacter cloacae* variant (39 h) and less stable *E. coli* enzyme (30 min) [70, 76]. However, activity modulation by various surfactants and chemical solvents [74] indicates that formulation optimization may be necessary for clinical applications. These biochemical characteristics, particularly the alkaline pH optimum, Mg²⁺ activation, and serum stability profile, suggest potential therapeutic advantages over conventional microbial L-asparaginases. Notably, the enzyme exhibited a serum half-life of 60 min, which is twice as long as *E. coli* L-asparaginase (30 min) [70], suggesting better adaptation to human serum’s physicochemical conditions. While this in vitro stability is promising, further studies are needed to evaluate its in vivo pharmacokinetics. For comparison, the *E. coli* type II enzyme shows an in vivo half-life of 1.24 days [77] despite its rapid in vitro inactivation, highlighting the complex relationship between in vitro and in vivo stability. The enzyme’s phosphate buffer half-life (3.8 h) and enhanced serum tolerance may offer clinical advantages. Its higher specific activity, combined with sustained stability in physiologically relevant conditions, could reduce dosing frequency while maintaining therapeutic efficacy. These properties position *S. acroporae* L-asparaginase as a potential alternative to current formulations, though in vivo studies are essential to validate its pharmacokinetic profile.

The enzyme exhibited maximum catalytic activity at a substrate concentration of 0.05 M L-asparagine. Kinetic analysis revealed a Michaelis constant (Km) of 0.007271 M and a maximum reaction velocity (Vmax) of 84.31 U ml⁻¹ min⁻¹, indicating high substrate affinity and robust catalytic efficiency [78]. The Km value, representing the substrate concentration at half-maximal velocity, reflects the enzyme-substrate binding affinity, lower values denote stronger binding. Our measured Km demonstrates superior binding affinity for L-asparagine compared to reported values for *E. coli* (0.058 mM) and *E. chrysanthemi* (0.015 mM) L-asparaginases [79]. This enhanced substrate affinity is particularly relevant for therapeutic applications, as the antitumor efficacy of L-asparaginase correlates directly with its binding affinity for asparagine. The kinetic parameters suggest this enzyme may offer advantages in the depletion of circulating asparagine, a critical factor in its anti-leukemic activity.

While conventional asparaginases often lack direct cytotoxic effects on tumor cells [80, 81], our study demonstrates that purified L-asparaginase from *S. acroporae* S4-41 exhibits significant dose-dependent cytotoxicity against multiple cancer cell lines, with IC50 values of 513 µg/mL (NB4), 737.4 µg/mL (HepG-2), and 711.5 µg/mL (MCF-7). This cytotoxic activity occurs through several interconnected mechanisms: asparagine depletion impairs protein synthesis and cell proliferation [82, 83]; reduced serine availability disrupts nucleic acid production [84]; metabolic interference occurs via conversion to oxaloacetate in the TCA cycle [85]; and mitochondrial dysfunction triggers apoptosis [86]. These findings align with reports by Amena et al. [36] regarding thermal optimization, as both *S. acroporae* and *Streptomyces gulbargensis* show peak activity at 40 °C.

The enzyme from a halophilic origin may confer therapeutic advantages, including enhanced stability under physiological conditions and improved serum compatibility compared to mesophilic counterparts. While the IC50 values are higher than fungal asparaginases (e.g., *Fusarium solani* at 3.66 µg/mL for leukemia cells), this marine-derived enzyme shows promise against solid tumors. Future studies should investigate in vivo efficacy, potential synergies with existing chemotherapies, and structure-function relationships to optimize tumor specificity.

To better contextualize the catalytic efficiency of the purified *S. acroporae* L-asparaginase, its kinetic parameters were compared with those reported from other microbial sources, including *E. coli*, *Erwinia carotovora*, *Bacillus*, and and *Streptomyces* species. The markedly lower Km observed for *S. acroporae* indicates superior substrate affinity, while its Vmax, though moderate, remains within a therapeutically relevant range. This comparison highlights the potential of extremophilic and coral-associated bacteria as valuable reservoirs of novel biocatalysts with unique kinetic properties (Table 5).

**Table 5:** Comparative kinetic parameters (Km and Vmax) of L-asparaginases from *Salinicola acroporae* and other microbial sources

Source organism	Km	Vmax	Notes/references
*Salinicola acroporae* (this study)	0.00727 mM	84.31 U/mL/min	Novel coral-associated halophilic strain; very low Km indicating strong substrate affinity; moderate Vmax but clinically relevant
*E. carotovora* (native)	7.14 M	0.05 mg/mL/min	Reported under pH 8.6, 35 °C conditions; unusually high Km suggests weak affinity [87]
*E. carotovora* (alternative report)	0.098 mM	1666.7 µmol/mg/min	Considerably lower Km; thiol activation enhances activity [88]
*E. coli* (pathogenic)	1.25 × 10 mM (≈ 12.5 mM)	2.5 × 10 M/min	Commonly used clinically; values vary with assay methods [89]
*E. coli* (recombinant)	0.5 mM	500 U/mg	Engineered enzyme with higher catalytic efficiency and stability [90]
*Bacillus megaterium*	2.0 × 10⁻⁴ M (0.2 mM)	1.198 mM/s	Reported as highly efficient bacterial source [91]
*Streptomyces* HB2AG	0.0021 M (2.1 mM)	714.3 µM/min	Actinomycete source with industrial potential [92]

## Conclusion

This study characterizes a novel glutaminase-free L-asparaginase from *Salinicola acroporae* S4-41 with significant therapeutic potential. The enzyme demonstrates exceptional substrate specificity, showing strong affinity for L-asparagine while exhibiting minimal glutaminase activity. Biochemical characterization revealed optimal activity at physiologically relevant conditions (pH 8.0, 40 °C) and remarkable stability in serum environments (60 min half-life at 37 °C), outperforming conventional *E. coli*-derived asparaginases. The enzyme’s halophilic nature confers additional advantages, including tolerance to saline conditions and resistance to various metal ions and solvents. Of particular significance is the enzyme’s demonstrated cytotoxicity against multiple cancer cell lines (NB4, HepG-2, and MCF-7), with IC50 values ranging from 513 to 737.4 µg/mL, while maintaining specificity that spares healthy cells. This anticancer activity stems from its ability to deplete asparagine pools, disrupt protein synthesis, nucleotide production, and mitochondrial function in malignant cells. Future study should focus on (1) increasing manufacturing efficiency by genetic engineering and nanoscale formulation, (2) assessing the In vivo efficacy and pharmacokinetics, and (3) investigating synergistic combinations with existing medicines.

## Data Availability

No datasets were generated or analysed during the current study.
